# Circulating miRNAs in genitourinary cancer: pioneering advances in early detection and diagnosis

**DOI:** 10.1016/j.jlb.2025.100296

**Published:** 2025-04-25

**Authors:** Annunziata Gaetana Cicatiello, Michele Musone, Silvestro Imperatore, Carlo Giulioni, Roberto La Rocca, Angelo Cafarelli, Francesco Del Giudice, Monica Dentice, Felice Crocetto

**Affiliations:** a Department of Clinical Medicine and Surgery, University of Naples “Federico II”, Naples, Italy; b Department of Neurosciences, Reproductive Sciences and Odontostomatology, University of Naples Federico II, Italy; c Casa di Cura Villa Igea, Via Maggini, 200, Ancona, Italy; d CEINGE – Biotecnologie Avanzate Scarl, Naples, Italy; e Department of Maternal Infant and Urologic Sciences, "Sapienza" University of Rome, Viale Regina Elena 324, 00161, Rome, Italy

**Keywords:** MicroRNA (miRNA), Liquid biopsy, Cancer diagnosis, Non-coding RNA, Gene expression regulation, Tumor biomarkers, Precision medicine, Urological cancers, Prostate cancer, Bladder cancer, Renal cell carcinoma, Testicular cancer, OncomiRs, Tumor suppressor miRNAs, Metastasis biomarkers, Artificial intelligence in oncology, Early cancer detection, Personalized cancer therapy, Circulating miRNAs, Non-invasive diagnostic tools

## Abstract

MicroRNAs (miRNAs) are noncoding, single-stranded RNAs molecules modulating gene expression thanks to their ability to bind mRNAs. Indeed, they regulate key processes for the proper functioning of the organism and affect the course of human pathologies. Altered expression of individual miRNAs has been shown in most human cancers, endowed with oncogenic or suppressor potential. Additionally, miRNAs have shown a promising potential as cancer markers. Not only miRNAs are present in various body fluids, such as serum and plasma, they also have high biological stability of miRNAs that in turn facilitates their detection. For these reasons, the use of miRNAs detection as non-invasive prognostic and predictive biomarkers in cancer provides new perspectives and strategies for personalized medicine and cancer therapy.

This review summarizes the current knowledge supporting the utility of miRNAs as novel early diagnostic and prognostic tool, with a special focus on the effectiveness of miRNAs of the liquid biopsies in the diagnosis of urogenital cancers. In conclusion, we rightly state that circulating miRNAs and therefore liquid biopsy have the potential to revolutionize the diagnosis, monitoring and even treatment of urological tumors, entering clinical practice as a more accurate, less invasive and more suitable diagnostic tool for patient follow-up compared to current methodologies. With the progress of scientific research and the implementation of advanced technologies that also exploit artificial intelligence, the future of oncological diagnosis, especially for urological tumors, could be increasingly oriented towards less invasive approaches and based on precision medicine, tailored for the specific patient.

## Graphical abstract

**Figure:**
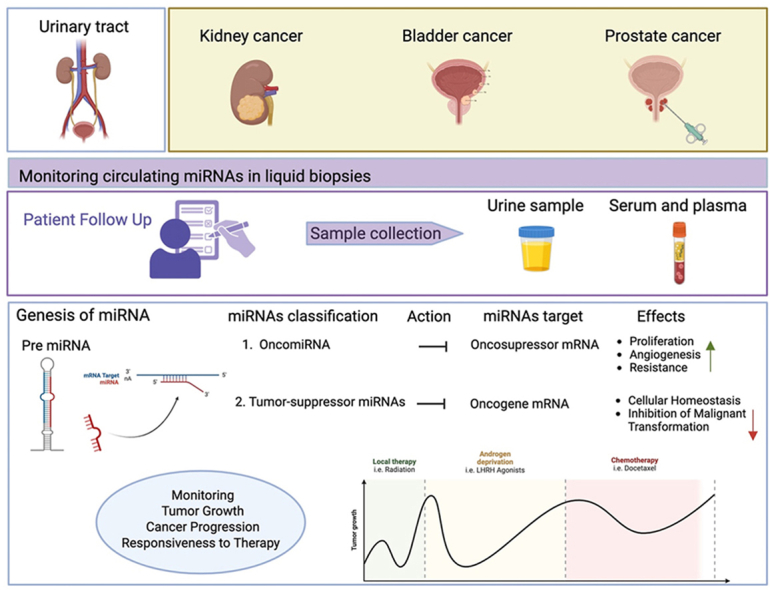

## 1 Introduction

### 1.1 MicroRNAs: master regulators of gene expression and cellular function

MicroRNAs (miRNAs) are small, non-coding RNA molecules that act as key regulators of gene expression at the post-transcriptional level. Typically ranging from 18 to 25 nucleotides in length, miRNAs exert their function by binding to complementary sequences on messenger RNA (mRNA), leading to either mRNA degradation or translational repression [1]. This mechanism allows miRNAs to fine-tune protein synthesis, ensuring precise control over cellular processes and maintaining homeostasis in various biological systems.

Since their initial discovery in Caenorhabditis elegans in 1993 [2], miRNAs have been increasingly recognized for their widespread role in fundamental biological functions. They regulate key pathways involved in cell differentiation, proliferation, apoptosis, immune responses, and metabolic homeostasis [3]. Acting as molecular switches, miRNAs coordinate complex gene expression networks, ensuring proper cellular function under physiological conditions while responding dynamically to environmental and developmental cues.

Beyond their role in cellular maintenance, miRNAs are integral to physiological adaptations and stress responses. They contribute to tissue development, organogenesis, and neurogenesis, playing crucial roles in processes such as muscle development [4], cardiovascular function [5] and neuronal plasticity [6]. Additionally, miRNAs are involved in immune regulation, modulating innate and adaptive immune responses by influencing cytokine signaling, T-cell differentiation, and inflammatory pathways [7,8].

The significance of miRNAs extends beyond human biology, as they are highly conserved across species and play an essential role in animal health and development [9]. In veterinary medicine, miRNA research is advancing our understanding of physiological processes in diverse species, offering valuable insights into evolutionary biology and potential applications in animal health management [10].

As research continues to unravel the complexity of miRNA regulatory networks, these molecules are being increasingly explored as potential biomarkers for disease detection and as targets for novel therapeutic interventions [11]. With advancements in high-throughput sequencing, bioinformatics, and synthetic biology, miRNAs are poised to transform our understanding of molecular biology, paving the way for innovative strategies in diagnostics, regenerative medicine, and personalized healthcare.

### 1.2 The role of MicroRNAs in cancer: from tumorigenesis to targeted therapy

The role of miRNAs extends beyond physiological functions to disease pathogenesis, particularly in cancer, where they modulate intricate gene expression networks involved in tumorigenesis [12]. In this context, miRNAs can function either as oncogenes (oncomiRs) or tumor suppressors, depending on their target genes and cellular context. OncomiRs promote cancer progression by downregulating tumor suppressor genes, leading to enhanced proliferation, angiogenesis, and resistance to apoptosis. Conversely, tumor-suppressor miRNAs inhibit oncogenic pathways, maintaining cellular homeostasis and preventing malignant transformation [13]. The alteration of miRNA expression is a hallmark of cancer, driven by genetic and epigenetic changes such as chromosomal deletions, amplifications, DNA methylation, and histone modifications [14]. Dysregulated miRNAs contribute to the six hallmarks of cancer, including sustained proliferative signaling, evasion of growth suppressors, resistance to cell death, replicative immortality, angiogenesis, and metastasis [15].

Certain oncomiRs, such as miR-21 and miR-155, are overexpressed in multiple cancers, promoting tumorigenesis by inhibiting tumor suppressors like PTEN and TP53 [16]. miR-21 is upregulated in glioblastoma, breast, and lung cancers, driving oncogenic pathways [17]. Conversely, tumor-suppressor miRNAs, such as let-7 and miR-34, target oncogenes like RAS and MYC, preventing uncontrolled cell proliferation [18]. However, their downregulation in tumors removes these critical restraints, leading to cancer progression.

Metastasis that represents the primary cause of cancer-related mortality is tightly regulated by specific miRNAs that influence epithelial-to-mesenchymal transition (EMT), extracellular matrix remodeling, and tumor cell migration. Several miRNAs play critical roles: miR-10b exerts a pro-Metastatic role, enhancing tumor invasion and metastasis by directly targeting HOXD10, a metastasis suppressor, leading to increased RhoC activation and cellular motility in breast cancer [19]. By contrast, miR-335 and miR-126 are metastasis-suppressing miRNAs inhibit metastatic dissemination by suppressing genes involved in cell motility and invasion. Loss of these miRNAs correlates with poor prognosis and aggressive tumor behavior.

One of the most promising aspects of miRNA research is their stability in bodily fluids such as blood, saliva, and urine, making them excellent candidates for liquid biopsies [19]. Unlike traditional tissue biopsies, miRNA-based liquid biopsies provide real-time, minimally invasive diagnostic tools for early cancer detection and disease monitoring (17).

Distinct miRNA expression profiles have been identified for different cancer types suggesting the possibility of defining a cancer-specific miRNA signature. For example, miR-141 in prostate cancer; miR-200c in colorectal cancer; miR-155 in breast cancer; miR-21 in lung cancer; miR-196a in pancreatic cancer [20]. These signatures can aid in early-stage diagnosis, prognosis, and treatment selection, providing valuable insights for precision medicine.

The potential of miRNA-based therapies has gained momentum, focusing on two primary approaches: oncomiR inhibition and tumor-Suppressor miRNA replacement. The oncomiR inhibition strategy aims to suppress oncogenic miRNAs using antisense oligonucleotides (anti-miRs) in order to restore tumor suppressor function. An example of this approach is the Anti-miR-21 therapy that enhances apoptosis in glioblastoma cells [21]. Instead, the tumor-suppressor miRNA replacement utilizes miRNA mimics to restore lost tumor-suppressive miRNAs. An application is miR-34 mimics effectively suppress tumor growth in lung cancer models. Combining miRNA therapies with chemotherapy, radiotherapy, or immunotherapy has shown synergistic effects, improved treatment efficacy and overcoming resistance [22]. Additionally, advancements in high-throughput sequencing, biosensors, and artificial intelligence have refined miRNA profiling, accelerating their integration into personalized oncology [23]. Despite the exciting potential of miRNA-based diagnostics and therapeutics, several challenges remain: i. Ensuring miRNAs reach their target tissues without degradation. ii. Minimizing off-target effects to avoid unintended gene interactions [13]. Developing reproducible and cost-effective diagnostic assays to standardize the detection methods. With ongoing research and technological advancements, miRNAs are poised to revolutionize cancer detection, prognosis, and treatment, offering new hope in the realm of precision medicine.

## 2 Effectiveness of micrornas for cancer diagnosis

In recent years, circulating microRNAs (miRNAs) and small non-coding RNA as molecules capable of regulating gene expression, have increasingly attracted the attention of the scientific community, especially for their role in cancer diagnosis. These small molecules, in fact, have proven to be promising biomarkers for numerous and different types of tumors, especially urological ones [24,25] such as prostate, bladder and renal cancer (Graphical Abstract). Currently, in clinical practice, the diagnosis of most urological tumors is based on invasive methods, such as tissue biopsies, which, although effective, still have significant intrinsic limitations: in addition to being painful and carrying risks of complications for patients, they are not always able to provide a complete picture of the disease due to tumor heterogeneity. Furthermore, performing repeated biopsies to monitor disease progression and to allow an active neoplastic survey, is often impractical. Faced with these often-insurmountable difficulties, scientific research has focused on finding non-invasive tests capable of providing precise information on the tumor and which therefore meet the needs that an increasingly demanding and precise clinical practice requires. This is where liquid biopsy comes into play, an innovative approach that allows the detection of tumor biomarkers - including miRNAs - in blood, urine or other biological fluids. Urological cancers, including those of the prostate, bladder, kidney, testicle, and adrenal gland, place a considerable burden on global health care systems. Early detection remains critical to improving patient outcomes, although current diagnostic tools are limited in both sensitivity and specificity [26,27]. For example, prostate cancer, the most commonly diagnosed cancer among men, screening relies primarily on measurement of prostate-specific antigen (PSA). However, PSA testing lacks specificity: elevated levels may also result from benign prostatic hyperplasia (BPH), inflammation, or recent medical procedures [28]. As a result, many patients undergo unnecessary biopsies, which carry risks and discomfort without providing decisive diagnostic benefit.

The situation is even more critical for other urinary cancers. Diagnosis of bladder cancer often requires invasive cystoscopy and has poor sensitivity for low-grade tumors with current urine cytology. Renal and adrenal tumors are often diagnosed incidentally and reliable early biomarkers are lacking. Testicular cancer, although more common among younger men, also suffers from diagnostic challenges: traditional serum markers such as AFP, hCG, and LDH are inadequate for early diagnosis in a substantial proportion of patients.

These limitations underscore the urgent need for minimally invasive, highly specific, and broadly applicable diagnostic tools. In this context, liquid biopsy, particularly by analyzing circulating microRNAs (miRNAs), has emerged as a promising solution. ImiRNAs are small noncoding RNA molecules that are stable in body fluids and can reflect the molecular characteristics of tumors [29]. They have shown potential not only for distinguishing malignant from benign conditions but also for risk stratification, treatment response prediction, and post-treatment surveillance.

Take, for example, circulating microRNAs miR-141 and miR-375: these have shown a higher specificity than PSA in detecting prostate cancer. Meanwhile, miR-21 and miR-221 are associated with tumor aggressiveness and therapy resistance [30]. The ability to detect these biomarkers with a simple blood or urine sample could revolutionize prostate cancer diagnosis, not only by reducing the number of unnecessary biopsies but also by improving risk stratification for patients, as well as our ability to decide ab initio on the necessary treatment for a given tumor. In bladder cancer, urinary miRNAs such as miR-126 and miR-200a are significantly downregulated, while miR-21 and miR-155 are overexpressed in aggressive forms of the disease and are implicated in tumor growth and treatment resistance [31], so measuring these important markers could also be essential to monitor disease progression and response to certain types of treatment. Similarly, in renal and adrenal tumors, distinct miRNA signatures, such as miR-210 and miR-483-5p, hold promise to differentiate between benign and malignant lesions. This review aims to summarize the current knowledge on the clinical utility of miRNAs in liquid biopsy, with a specific focus on their diagnostic application across the spectrum of urological malignancies.

By highlighting unmet needs and demonstrating advances in miRNA-based diagnostics, we set the stage for a paradigm shift toward precision oncology, driven by noninvasive, molecularly targeted approaches.

## 3 The diagnostic use of micrornas in liquid biopsies

Many liquid biopsy examinations are accessible nowadays with prostate cancer screening in mind. Among those, the SelectMDx test analyses a series of miRNAs found in urine. The point is to pinpoint the presence of clinically relevant tumors, minimizing the demand for regular biopsies [32]. Another approach, the ExoDx Prostate Test, works with the examination of urinary exosomes, which harbor miRNAs. This permits to tell the difference between harmless and dangerous tumors. Liquid biopsy is therefore turning into a vital tool for prostate cancer diagnosis [33,34], which promises to bring more accurate and less invasive detections, which, by consequence, reduces the number of unnecessary operations [35,36].

Nevertheless, while urinary tests are a considerable advantage, the circulating miRNAs derived from blood are a vital part of liquid biopsy, becoming increasingly crucial in clinic. Many researches have showed how effective blood plasma or serum miRNAs are in acting as non-invasive biomarkers for prostate cancer. To exemplify, miR-141 and miR-375 increase considerably in the serum of patients with advanced prostate cancer. They have also been associated with metastatic illnesses and tumor load [30]. Additionally, miR-21, one of the most researched oncomiRs, is increased in prostate cancer, related to treatment resistance, particularly in androgen deprivation therapy [31]. These blood-based indicators can be a helpful addition to PSA screenings and improve the accuracy of a diagnosis, especially in uncertain cases or during follow-up.

Risk stratification has also been proposed to be performed via the usage of blood-based miRNAs. Certain groups of miRNAs, including miR-145, miR-221, and miR-182, have given interesting results in discerning low-risk and high-risk prostate cancer. This might help with making decisions clinically in active surveillance versus active treatment. Those same miRNAs, detectable via a simple blood draw, allow a real-time method to track the illness, facilitating the continuous assessment of treatment response, and the identification of early stages of recurrence.

In a second prominent area in which miRNAs are used there is bladder cancer. The cancer's rate of recurrence is elevated. As a consequence, a close and continuous follow-up is required. The cystoscopy procedure has become the main technique for the detection of bladder cancer in today's clinical practice. However, cystoscopy is an intrusive and poorly tolerated examination, and urinary cytology can only detect low-grade tumors to a certain extent [37,38]. Consequently, the discovery of urinary biomarkers, mainly miRNAs, turns out to be a large step forward. Consider, for instance, miR-126 and miR-200a; their levels have been proven to be low in patients with bladder cancer, while miR-21 and miR-155 are usually overexpressed and linked to the aggressive tumor types [39,40]. Liquid biopsy examinations, utilizing urine, have already shown excellent results in terms of diagnostic accuracy and specificity. Tools such as CxBladder™, that analyze a set of urinary biomarkers, have demonstrated outstanding performance and could soon be integrated regularly into clinical practice, so minimizing the dependency on cystoscopy [41]. Meanwhile, the diagnostic and prognostic use of circulating miRNAs in blood samples has also gained popularity within the context of bladder cancer. Blood-based tests provide the advantage of systematic biomarker detection, especially beneficial with high-grade or muscle-invasive tumors. The levels of serum miR-21 and miR-155, for example, have correlated with the disease's stage and prognosis. Additional notable candidates include miR-192, miR-200c, and miR-214 [42]. Together they have provided excellent results regarding diagnostic accuracy in distinguishing those affected by bladder cancer from the healthy individuals. These circulating miRNAs can also become a way to monitor treatment responses, particularly in those undergoing immunotherapy or BCG treatment. Moreover, they could help reveal any initial recurrence indicators, thus playing a significant role in patient follow-up. Overall, all of the discoveries discussed above put the stress on how essential it is to integrate the two assays of miRNAs (the ones using urine and blood-derived) into clinical algorithms for prostate and bladder cancers. Using the assays combined might enhance the early stages of detection, guide the decisions regarding treatment, and personalize the follow-up approaches in a way that is minimally invasive and patient-centered [43,44].

As regards renal cell carcinoma, however, this represents an even more complex diagnostic challenge. In fact, renal cell carcinoma is often discovered by chance during imaging tests performed for other reasons [45], and currently there are no reliable biomarkers for the early diagnosis of this tumor. In this case too, liquid biopsy with miRNA research could fill the diagnostic gap that has always permeated this tumor [46,47]. Recent studies have shown that miR-210 is one of the most frequently overexpressed miRNAs in patients with renal cancer, and miR-21 and miR-155 have also been shown to be associated with the most aggressive forms of the disease [48,49]. Thanks to liquid biopsy, in the future it may be possible to distinguish between benign and malignant renal masses without having to resort to invasive biopsies [50,51] or, worse, surgical interventions aimed at removing the lesion. In this way, the clinical management of patients could be exponentially improved, also facilitating post-operative monitoring and subsequent [52,53] follow-up to identify any neoplastic recurrences early [54,55].

Adrenal tumors diagnosis can also benefit from microRNA detection. Indeed, the diagnosis of these tumors is extremely complex, mainly in asymptomatic ones, and is often made incidentally, during diagnostic tests to search for other pathologies. For this reason, the studies conducted on microRNAs are extremely interesting, in which it was demonstrated that five microRNAs (hsa-miR-100, hsa-miR-181b, hsa-miR-184, hsa-miR-210 and hsa-miR-483-5p) showed a statistically significant overexpression in adrenocortical cancer vs adenoma, thus also helping in identifying the cause of the disease and avoiding unnecessary surgical interventions. These studies are fundamental in the perspective of an increasingly minimally invasive medicine that aims to make a diagnosis even with a simple blood sample [56,57].

In the field of the upper excretory tract, interesting studies have been carried out regarding the correlation between urothelial tumors and the contribution of microRNAs to their resolution. In fact, researchers are trying to demonstrate how the miRNA 145-p, implicated in the receptor down-regulation of 5-aminoimidazole 4-carboxamide ribonucleotide formyltransferase/inositol monophosphate cyclohydrolase (ATIC) can be a biomarker of the possible response to the disease of the human body. The same goes for the microRNA 299p-3p which, by limiting the receptor expression of VEGFA [58], can reduce tumor angiogenesis. Fundamental studies as they make us understand how microRNAs can also be used to evaluate the progression of the disease.

Testicular cancer is one of the most common cancer among young men, mainly the ones between the ages of 15 and 35. Despite its relative rarity in comparison to other cancer types, the incidence of testicular cancer has been heavily increasing over the last years [59]. The early detection and effective treatment of testicular cancer are fundamental for improving patient outcomes and survival rates. Actually the diagnosis of testicular cancer is based on clinical examination, imaging techniques, and serum markers like alpha-fetoprotein (AFP), human chorionic gonadotropin (hCG), and lactate dehydrogenase (LDH) [60]. But these biomarkers have limitations, particularly in the early stages of the disease. In this situation the MiRnas are fundamental because their dysregulation has been implicated in various stages of cancer [61], including initiation, as mentioned earlier, progression, and metastasis [62,63]. The early detection of testicular cancer although remains an important clinical challenge.In fact traditional tumor markers such as AFP, hCG, and LDH are useful for monitoring the progression of testicular cancer [64] but it's not always possbile to use them for early diagnosis [65]. Many studies and meta-analysis have identified some miRNAs that are expressed in different ways in the blood, serum, and semen of testicular cancer patients. For example, the microRna-371-3 is heavily upregulated in the serum of patients with testicular germ cell tumors, showing potential as a highly sensitive biomarker for detecting testicular cancer, also in the early stages of the disease [66]. Furthermore, also other microRnas have been identified as potential diagnostic biomarker as miR-141, miR-146a, and miR-204 [67]. Other studies instead tried to prove the ability of microRNAs to distinguish between different subtypes of testicular cancer like miR-372 and miR-373 that have been shown to be upregulated in non-seminomatous tumors. The importance of this discover is that these microRNAs could potentially be used to differentiate between seminomas and non-seminomas, helping the choose of the best medical treatment and giving insights about prognosis [68]. Then, while imaging techniques and serum markers are commonly used to assess therapeutic outcomes, microRNAs could offer a more sensitive and specific alternative for diagnosis in non-invasive way. In conclusion, microRNAs represent a promising discovery as biomarkers for testicular cancer. Their stability in biological fluids, ability to reflect the molecular changes associated with cancer, and potential to differentiate between cancer subtypes make them the best non-invasive diagnostic tools [69,70].

## 4 Conclusions

Liquid biopsy represents one of the most promising innovations in cancer diagnosis for a whole series of reasons. In fact, unlike traditional biopsies, in addition to being atraumatic and free of complications and discomfort for patients, it also gives us the possibility of monitoring the evolution of the disease in real time through a simple blood or urine sample, without risks for the patient. Thanks to their high tumor specificity and their stability in biological fluids, miRNAs are establishing themselves as ideal biomarkers to improve early diagnosis, reduce the number of invasive procedures and optimize the therapeutic management of cancer patients both in the diagnostic phase and in the subsequent oncological follow-up. However, for these tests to become part of clinical practice, it will be necessary to standardize the detection methodologies and conduct large-scale, quality studies to validate their efficacy and safety. Furthermore, in the future, integration with software that exploits artificial intelligence could further improve the interpretation of data, paving the way for increasingly personalized and precise medicine.

**Table:** 

miRNA	Function and Role	Cancer Type	Biofluid	Expression	Reference
miR-21	OncomiR, associated with tumor growth and treatment resistance	Prostate, bladder, kidney	Blood, Urine	Upregulated	[30]
miR-221	Involved in tumor proliferation and drug resistance	Prostate	Blood	Upregulated	[30]
miR-375	More reliable than PSA in distinguishing cancer from benign conditions	Prostate	Blood, Plasma	Upregulated	[30]
miR-141	Biomarker for advanced-stage diagnosis	Prostate, testicle	Serum, Plasma	Upregulated	[20]
miR-126	Reduced in bladder cancer patients	Bladder	Urine, Serum	Downregulated	[19]
miR-200a	Reduced in bladder cancer patients	Bladder	Urine	Downregulated	[21]
miR-155	Associated with rapid tumor growth	Bladder	Serum, Urine	Upregulated	[16]
miR-210	Diagnostic biomarker	Kidney, adrenal gland	Serum	Upregulated	[56,57]
miR-483-5p	Differentiates between adenoma and carcinoma	Adrenal gland	Blood	Upregulated	[56,57]
miR-100	Overexpressed in adrenocortical carcinoma	Adrenal gland	Serum	Upregulated	[56,57]
miR-181b	Differential marker between benign and malignant tumors	Adrenal gland	Serum	Upregulated	[56,57]
miR-184	Overexpressed in adrenocortical carcinoma	Adrenal gland	Blood	Upregulated	[56,57]
miR-145	Associated with tumor suppression	Prostate	Blood	Downregulated	[37,38]
miR-299-3p	Reduces tumor angiogenesis by limiting VEGFA	Upper urinary tract	Serum	Downregulated	[47]
miR-371-3p	Sensitive biomarker for testicular cancer	Testicle	Serum	Upregulated	[64]
miR-372	Upregulated in non-seminomatous tumors	Testicle	Serum	Upregulated	[68]
miR-373	Upregulated in non-seminomatous tumors	Testicle	Serum	Upregulated	[68]
miR-204	Potential diagnostic biomarker	Testicle	Serum	Upregulated	[67]
miR-146a	Possible biomarker for testicular cancer	Testicle	Serum	Upregulated	[33]
miR-182	Potential marker for prostate cancer	Prostate	Serum	Upregulated	[30]
miR-4286	Investigated for role in prostate cancer diagnosis	Prostate	Serum	Inconclusive	[28]
miR-30c	Proposed as a diagnostic marker	Prostate	Plasma	Upregulated	[37]

## Approval ethical

This study did not involve experiments on human participants or animals conducted by any of the authors. All data discussed in this article were obtained from previously published studies, which had received appropriate ethical approval. For studies involving human subjects, all referenced works adhered to the ethical standards of the institutional and/or national research committee and the 1964 Helsinki Declaration and its later amendments or comparable ethical standards. In cases where patient data were utilized, they were anonymized to ensure confidentiality. No new clinical samples or patient-related interventions were performed specifically for this study. Therefore, ethical approval was not required.

## Declaration of competing interest

The authors declare the following financial interests/personal relationships which may be considered as potential competing interests:Felice Crocetto reports was provided by University of Naples Federico II. If there are other authors, they declare that they have no known competing financial interests or personal relationships that could have appeared to influence the work reported in this paper.
